# A Novel Prognostic Signature Based on Glioma Essential Ferroptosis-Related Genes Predicts Clinical Outcomes and Indicates Treatment in Glioma

**DOI:** 10.3389/fonc.2022.897702

**Published:** 2022-06-10

**Authors:** Debo Yun, Xuya Wang, Wenbo Wang, Xiao Ren, Jiabo Li, Xisen Wang, Jianshen Liang, Jie Liu, Jikang Fan, Xiude Ren, Hao Zhang, Guanjie Shang, Jingzhang Sun, Lei Chen, Tao Li, Chen Zhang, Shengping Yu, Xuejun Yang

**Affiliations:** ^1^Department of Neurosurgery, Tianjin Medical University General Hospital, Tianjin, China; ^2^Laboratory of Neuro-oncology, Tianjin Neurological Institute, Tianjin, China; ^3^Department of Neurosurgery, Nanchong Central Hospital, Nanchong, China; ^4^Department of Neurosurgery, Beijing Tsinghua Changgung Hospital, Beijing, China

**Keywords:** glioma, ferroptosis, cancer essential genes, LASSO analysis, risk model, clinical outcomes, drug screening

## Abstract

**Background:**

Ferroptosis is a form of programmed cell death (PCD) that has been implicated in cancer progression, although the specific mechanism is not known. Here, we used the latest DepMap release CRISPR data to identify the essential ferroptosis-related genes (FRGs) in glioma and their role in patient outcomes.

**Methods:**

RNA-seq and clinical information on glioma cases were obtained from the Chinese Glioma Genome Atlas (CGGA) and The Cancer Genome Atlas (TCGA). FRGs were obtained from the FerrDb database. CRISPR-screened essential genes (CSEGs) in glioma cell lines were downloaded from the DepMap portal. A series of bioinformatic and machine learning approaches were combined to establish FRG signatures to predict overall survival (OS) in glioma patients. In addition, pathways analysis was used to identify the functional roles of FRGs. Somatic mutation, immune cell infiltration, and immune checkpoint gene expression were analyzed within the risk subgroups. Finally, compounds for reversing high-risk gene signatures were predicted using the GDSC and L1000 datasets.

**Results:**

Seven FRGs (ISCU, NFS1, MTOR, EIF2S1, HSPA5, AURKA, RPL8) were included in the model and the model was found to have good prognostic value (p < 0.001) in both training and validation groups. The risk score was found to be an independent prognostic factor and the model had good efficacy. Subgroup analysis using clinical parameters demonstrated the general applicability of the model. The nomogram indicated that the model could effectively predict 12-, 36-, and 60-months OS and progression-free interval (PFI). The results showed the presence of more aggressive phenotypes (lower numbers of IDH mutations, higher numbers of EGFR and PTEN mutations, greater infiltration of immune suppressive cells, and higher expression of immune checkpoint inhibitors) in the high-risk group. The signaling pathways enriched closely related to the cell cycle and DNA damage repair. Drug predictions showed that patients with higher risk scores may benefit from treatment with RTK pathway inhibitors, including compounds that inhibit RTKs directly or indirectly by targeting downstream PI3K or MAPK pathways.

**Conclusion:**

In summary, the proposed cancer essential FRG signature predicts survival and treatment response in glioma.

## Introduction

Gliomas are common primary malignant tumors of the central nervous system (1), representing nearly half of all primary intracranial neoplasms (2) and 80% of all malignant brain tumors (3). At present, the main treatment strategy for gliomas throughout the world is surgery (4) followed by postoperative adjuvant radiotherapy and chemotherapy (5, 6). Despite therapeutic advances, glioma patients have a poor prognosis (7), especially those with high-grade tumors (8), due to high levels of tumor cell diversity, proliferation, and metastasis (9). It has been hoped that the use of molecular markers may improve glioma characterization and predict survival (10, 11) but, thus far, the results, including those of clinical trials, have been disappointing (12). The identification of markers and potential targets that can be used for prediction and treatment is thus important, not only for managing the cancer but also for drug discovery.

Ferroptosis is a recently identified form of programmed cell death that is dependent on iron (13, 14). It has been linked with the outcomes of several cancers, including hepatocellular carcinoma, breast cancer, renal cell carcinoma, lung squamous cell carcinoma, and pancreatic carcinoma (15–19). Invasive and metastatic tumor cells have been found to be susceptible to ferroptosis (20) suggesting that targeting the process may be useful for treating cancer. However, there is limited information on the feasibility of targeting FRGs in glioma. The use of CRISPR-Cas9 knockout screening can elucidate relationships between genotype and phenotype through ablation of gene expression on a genome-wide scale and the consequent phenotypic alterations, for the specific identification of genes that could be targeted for inducing tumor growth inhibition or death. Our aim was to combine the CRISPR data to construct a novel prognostic signature based on the identified glioma-essential ferroptosis-related genes to specifically predict clinical outcomes and, importantly, to suggest directions for gene-targeted therapy.

## Material and Methods

### Data Collection and Preprocessing

**Figure 1:**
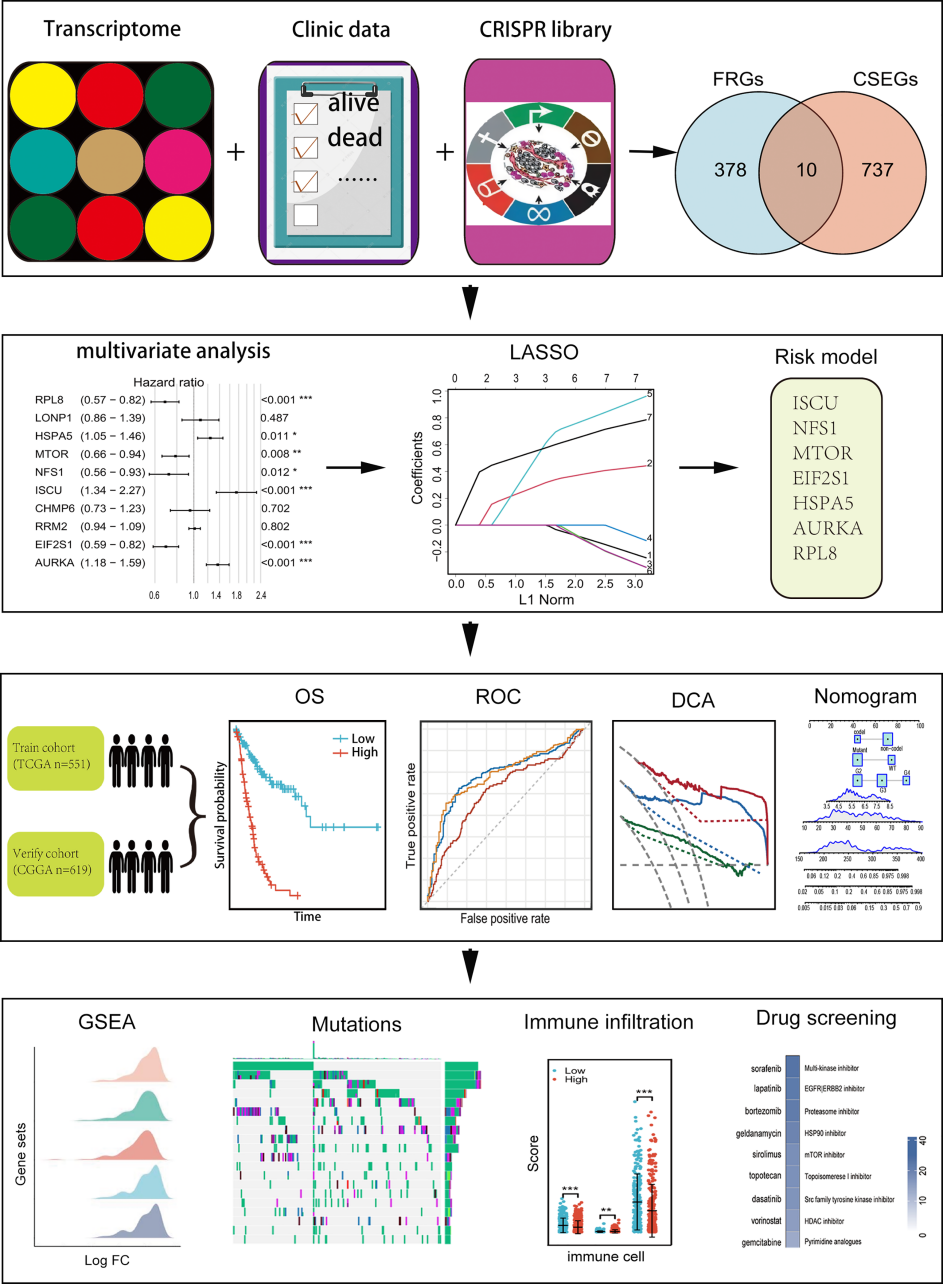
Flow chart showing the design of the study.

Clinical and RNA-seq transcripts per million reads (TPM) data were obtained from TCGA (https://xenabrowser.net) and CGGA-693 (http://www.cgga.org.cn). Patients with complete clinical data (age, sex, vital status, OS, PFI, IDH status, 1p-19q status, and WHO grade) were included. In all, 1170 patients were included, representing 551 in the training cohort and 619 in the validation cohort (**Table 1**). Ethical approval was waived as the study followed the TCGA and CGGA regulations for accessing data. Information on 388 FRGs was acquired from FerrDb (http://www.zhounan.org/ferrdb/) (**Table S1**) (21) and genome-wide CRISPR information on glioma cells was obtained from DepMap (https://depmap.org/portal/download/). The CERES algorithm (22) was used for the determination of dependency scores. First, genes with CERES scores below -1 in more than 75% of glioma cell lines were included. Many of these genes with known housekeeping functions that did not represent feasible targets were excluded, leaving 747 genes that were necessary for glioma cell survival *in vitro* (**Table S2**) and were termed glioma essential genes. Potentially targetable genes were identified as those falling into both FRG and CSEG categories. The flow chart of the study is illustrated in **Figure 1**.

**Table 1:** The clinical features of TCGA cohort and CGGA cohort.

	TCGA cohort	CGGA cohort
Characteristic	N = 551	N = 619
Age		
Median	47.21	43.44
Gender		
Male	313	356
Female	238	263
Grade		
Grage 2	209	173
Grage 3	232	231
Grage 4	110	215
IDH-status		
IDH_WT	188	258
IDH_Mut	363	316
1p/19q co-deletion		
Non-codel	403	427
Codel	148	128
Vital status		
Alive	365	296
Dead	186	323

### Construction and Verification of Risk Models

Before establishing the model, multivariate regression analysis was performed to identify potential genes related to prognosis (*p* < 0.05). Suitable candidate genes were identified as those with a minimal partial likelihood of deviance in LASSO regression and were used to construct the prognostic model (23). Risk scores were calculated as the sum of the products of regression coefficients and the expression of the individual genes. A cut-off value, represented by the median risk score, was used to separate both cohorts into high- and low-risk groups. The prognostic efficacy of the signature was assessed by analyzing survival. Time-dependent receiver operating characteristic (tROC) curves were used to calculate the area under the curve (AUC) for the 1-, 3-, and 5-year OS by using the R package “survivalROC” (24). Analysis of clinical correlates was performed between expression of cancer-essential FRGs and clinical characteristics using the R package “ggplot2” (25).

### Somatic Mutation, Immune Microenvironment and Function Enrichment Analyses

Mutation data were downloaded and visualized using the “maftools” R package to determine the somatic mutation landscape of glioma patients in the TCGA database, identifying distinct ferroptosis-related subtypes (26). Immune infiltration scores of glioma patients in the TCGA were downloaded from TIMER2.0 (http://timer.cistrome.org/) (27) and the patients were divided into high- and low-risk groups. The R packages “ComplexHeatmap” (28) and “ggplot2” were used to analyze and visualize the CIBERSORT algorithm data. Differentially expressed genes (DEGs) were identified using the median risk score as a threshold and the expression of the DEGs was analyzed using the R package “DESeq2” (29). Gene Set Enrichment Analysis (GSEA) was conducted on the TCGA cohort to examine the biological functions and pathways of the cancer-essential FRGs using the “clusterProfiler4.0” package in R (30).

### Nomogram Construction and Verification

The nomogram for glioma prognostic prediction was established using risk scores and clinical features (31). The accuracy and discriminative ability of the nomogram were assessed by calibration curves. Decision curve analysis (DCA) using the “ggDCA” and “stdca” packages (32) in R was performed to determine the survival net benefits at one, three, and five years for both OS and PFI (33).

### Identification of Potential Compounds

Spearman correlation analysis was used to identify compounds negatively associated with the AUC values and glioma cell FRG scores using the GDSC1 and GDSC2 drug response datasets (https://www.cancerrxgene.org) (34) using Spearman’s r < −0.30 and P-values < 0.05 for GDSC1 or <0.1 for GDSC2. DEG expression between the high- and low-risk groups was analyzed. The 150 most upregulated or downregulated genes in the high-risk group were used for further analysis. The relationships of 978 compounds with these 150 genes were examined in CLUE (summary connectivity score) (https://clue.io/repurposing) (35) and the genes were The 150 genes were then analyzed using iLINCS (http://www.ilincs.org/ilincs/) “Pharmacogenomics transcriptional signatures” (36) connectivity algorithms.

### Statistical Analysis

R version 4.0.2 was used for all analyses. Differences in normally distributed data were analyzed by unpaired t-tests, and those in non-normally distributed data by the Wilcoxon test. P-values <0.05 were considered significant.

## Results

### Construction and Verification of Risk Models

**Figure 2:**
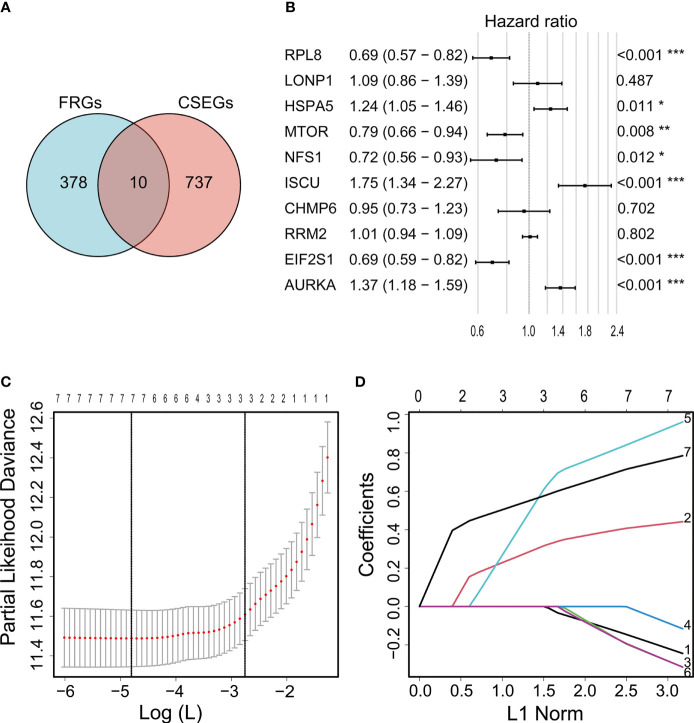
Venn diagram showing overlap of 10 genes between the CSEGs and FRGs **(A)**. Top prognosis-associated candidate genes identified by Cox regression **(B)**. Re-filtering of genes in **(B)** by LASSO **(C, D)**. *p < 0.05, **p < 0.01, ***p < 0.001.

Ten genes (ISCU, NFS1, MTOR, EIF2S1, HSPA5, AURKA, RPL8, LONP1, RRM2, and CHMP6) that fell into both the FRG and CSEG categories were used for further study (**Figure 2A**). Multivariate Cox regression showed that seven cancer-essential FRGs (ISCU, NFS1, MTOR, EIF2S1, HSPA5, AURKA, and RPL8) were significantly associated (*p*<0.05) with OS (**Figure 2B**). LASSO was then applied to reduce the number of these genes. As the partial likelihood was minimal, all the genes were retained and used for the construction of the risk model (**Figures 2C, D**). The risk score of the training cohort was calculated as 0.4181306×expression of HSPA5-0.2335796×expression of MTOR-0.0372456 ×expression of NFS1 +0.8788312×expression of ISCU-0.2320588×expression of EIF2S1+ 0.7371187×expression of AURKA-0.1765609×expression of RPL8.

### Prognostic Assessment Using the Risk Model

**Figure 3:**
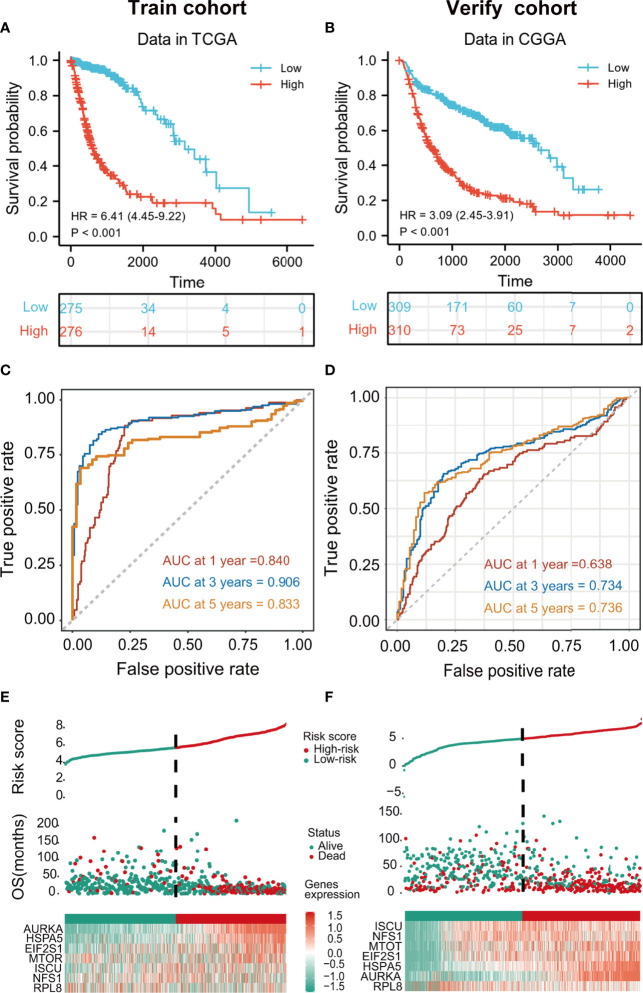
Kaplan-Meier curves showing risk scores of FRG signature genes in both training and validation cohorts (p < 0.001) **(A, B)**. tROC curves for one-, three- and five-year survival **(C, D)**. Heatmaps of risk score rankings, survival, and levels of cancer-essential FRGs in the training and validation cohorts **(E, F)**.

Patients in the training cohort were assigned to high- and low-risk groups according to the median risk score. Kaplan-Meier curves showed that members of the high-risk groups had significantly lower OS (p<0.001 in both cohorts) (**Figures 3A, B**). The AUCs for one-, three-, and five-year OS were 0.840, 0.906, and 0.833, respectively, in the training set (**Figure 3C**) and 0.638, 0.734, and 0.736, respectively, in the validation set (**Figure 3D**). The risk scores were then ranked in relation to OS and the levels of the seven cancer-essential FRGs were determined in the low- and high-risk groups, as shown in the heatmaps (**Figures 3E, F**). This indicated that a higher risk score was associated with lower OS for both groups, levels of the AURKA, EIF2S1, and HSPA5 genes were elevated in high-risk individuals in the training set, and all seven genes were strongly expressed in the high-risk category of the validation cohort.

### Relationships Between Risk Model Gene Levels and Clinical Characteristics

**Figure 4:**
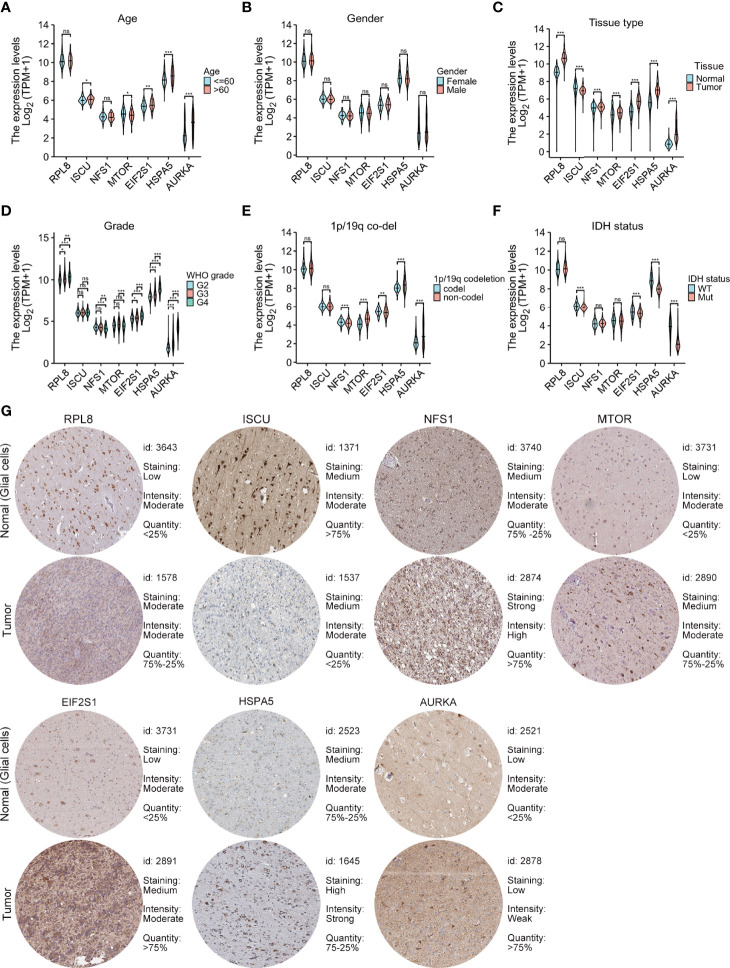
Relationships between the levels of the seven cancer-essential FRGs and clinical features. Age **(A)**, sex **(B)**, normal versus tumor tissue **(C)**, WHO grade **(D)**, 1p/19q co-deletion status **(E)**, and IDH status **(F)**. Immunohistochemistry showing the protein expression of risk model genes in normal and tumor specimens of The Human Protein Atlas **(G)**. ns, p≥0.05, *p < 0.05, **p < 0.01, ***p < 0.001.

m_sc_clean_body_s.tgaThe relationships between the levels of the seven signature genes and the clinical features of glioma patients from TCGA were investigated. The expression levels of these genes in tumor tissue were significantly different in comparison with those in normal tissues (**Figure 4C**). The levels of AURKA, EIF2S1, and HSPA5 were significantly associated with age (**Figure 4A**), grade (**Figure 4D**), the 1p/19q codeletion (**Figure 4E**), and isocitrate dehydrogenase (IDH) status (**Figure 4F**). The risk model genes were not related to sex (**Figure 4B**). In addition, immunohistochemical data on the risk model genes in normal and tumor specimens were downloaded from The Human Protein Atlas (https://www.proteinatlas.org/) for further verification (**Figure 4G**).

### Prognostic Value of the Risk Model for Subgroups of Clinical Classifications

**Figure 5:**
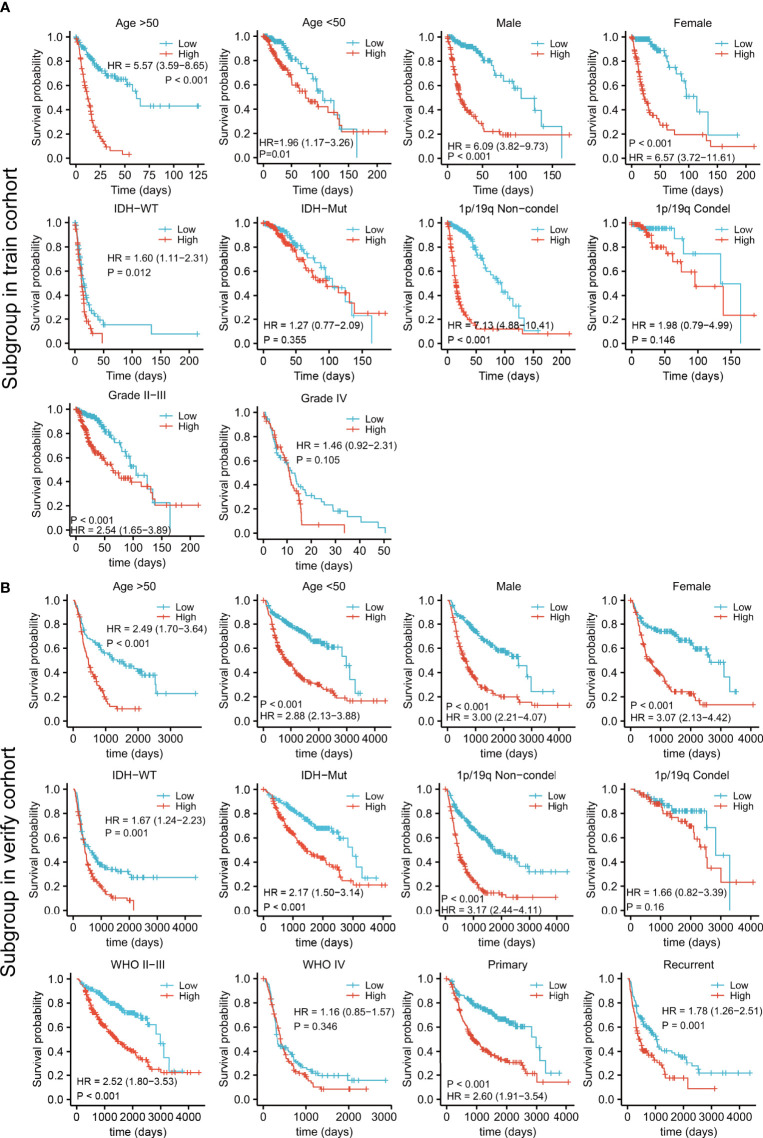
Survival analysis by the risk model in relation to subgroups of clinical features in the training **(A)** and validation cohorts **(B)**.

Patients in both the training and validation cohorts were divided into subgroups according to clinical features, including the age, sex, IDH status, 1p/19q con-deletion status, and WHO classification of the TCGA (**Figure 5A**) and the CGGA (**Figure 5B**) patients, as well as the primary/recurrent tumor types of the CGGA patients (**Figure 5B**). The subgroups were assessed in terms of median risk scores and survival curves were compiled for each of the clinical features. The results showed that the risk model was effective for these factors, except for patients with IDH-mut in the training cohort (**Figure 5A**) and those with the 1p/19q con-deletion and WHO IV in both the training and validation groups (**Figures 5A, B**).

### Comprehensive Analyses of Somatic Mutations, Immune Cell Infiltration, and Immune Checkpoint Expression in the Different Risk Groups

**Figure 6:**
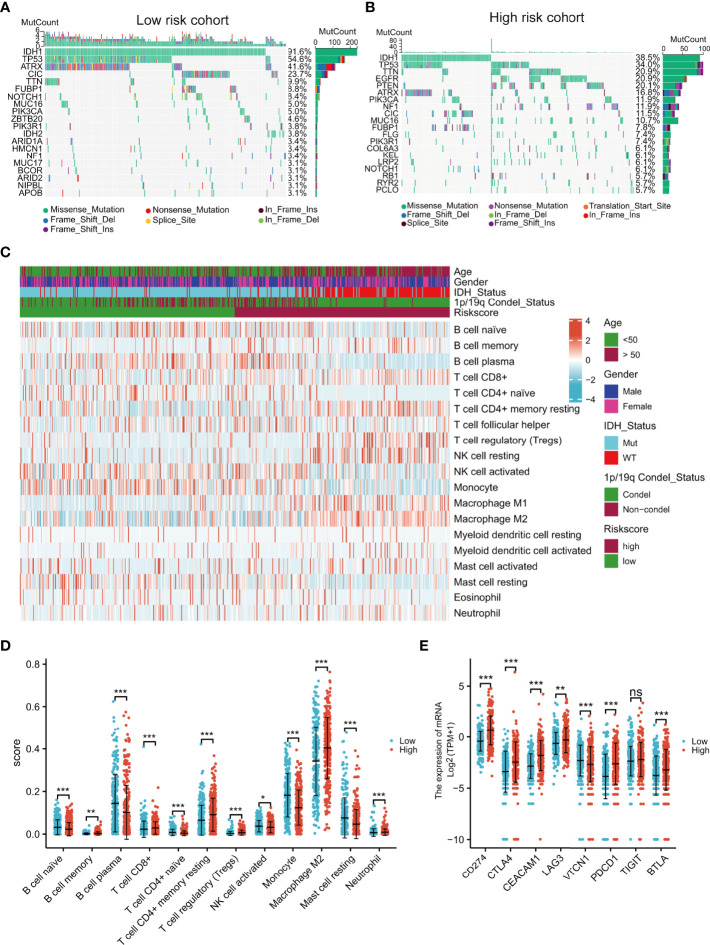
Comprehensive analyses of genomic alterations, immune cell infiltration, and immune checkpoint expression between the different risk groups. Distribution of sex, age, IDH status, 1p/19q condel status, and the top 20 most frequently mutated genes are illustrated for each cohort **(A, B)**. Heatmap showing the CIBERSORT scores of different immune cell distributions in the different subgroups **(C)**. Dot plot showing immune cell CIBERSORT scores and the expression levels of immune checkpoint gene in the high- and low-risk groups **(D, E)**. ns, no significance,*p < 0.05, **p < 0.01, ***p < 0.001.

Intuitively, the landscapes of somatic genetic alterations provided the 20 most frequently mutated genes in each risk cohort of TCGA samples. The results showed that 91.6% of patients in the low-risk group had IDH mutations, in contrast to only 38.5% in the high-risk group (**Figures 6A, B**), while PTEN and EGFR had higher rates of mutation in the high-risk group (**Figure 6B**). The immune cell infiltration scores of each sample were estimated using the CIBERSORT algorithm. Notably, the levels of regulatory T cells (Tregs) and M2 macrophages were higher in the high-risk group (**Figures 6C**, **D**) and the expression of immune checkpoint genes (CD274, CTLA4, CEACAM1, LAG3, TIGIT, PDCD1, and BTLA) were also higher in the high-risk group (**Figure 6E**). These results indicated that high-risk patients may be sensitive to PTEN or EGFR inhibition or the blocking of immune checkpoint gene expression.

### Construction and Verification of Nomogram

**Figure 7:**
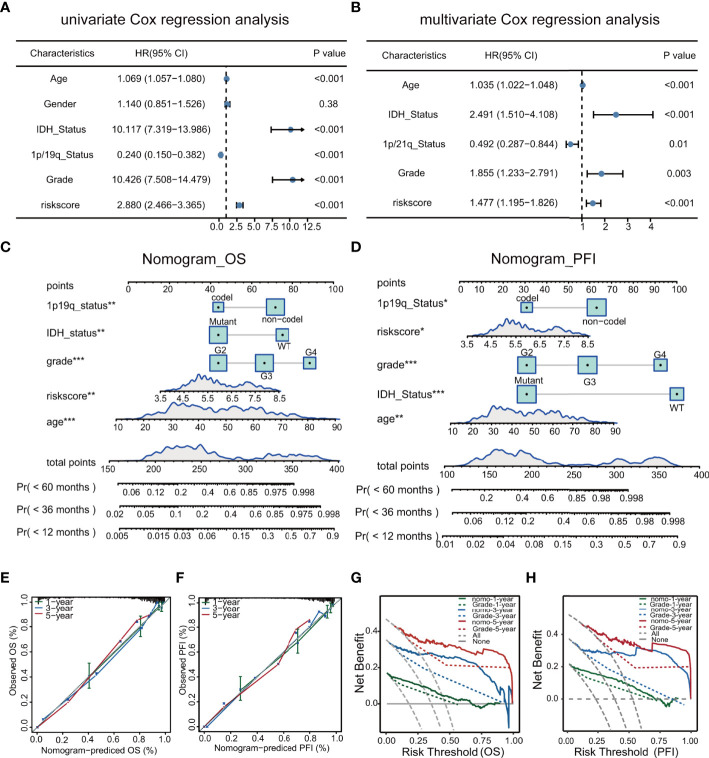
Univariate and multivariate analyses of clinical features in relation to prognosis **(A, B)**. Nomograms for prediction of OS and PFI **(C, D)**. Nomogram calibration using OS and PFI for predicted and actual one- three-, and five-year outcomes **(E, F)**. DCA of nomograms for OS and PFI for one- three-, and five-year survival **(G, H)**.

Univariate and multivariate analyses demonstrated that the risk score and clinical characteristics (age, grade, IDH status, and 1p/19q status) were risk factors linked to glioma prognosis in TCGA.(**Figures 7A, B**). The risk score was added to the nomogram prediction model for accurately predicting 12-, 36- and 60-months OS (**Figure 7C**) and PFI (**Figures 7D**). The calibration plots showed that the predicted and actual one- three- and five-year OS and PFI approximated well, indicating the good performance of the nomogram in comparison with an ideal model (**Figures 7E, F**). The clinical value of the nomogram was confirmed by DCA (**Figures 7G, H**).

### Gene Set Enrichment Analysis to Reveal the Functional Roles of Essential FRGs in Glioma

**Figure 8:**
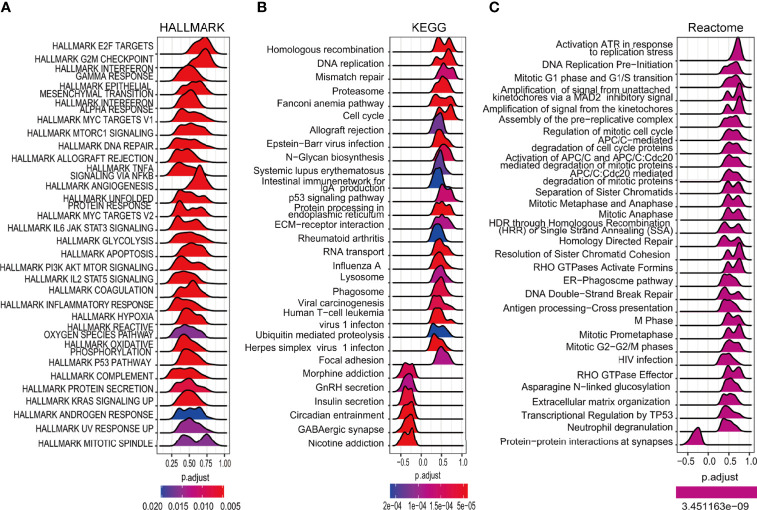
Enrichment analysis of cancer-essential FRG signature genes in the TCGA cohort. The top 30 gene sets of HALLMARK **(A)**, KEGG **(B)**, and Reactome **(C)**.

The expression of the DEGs in the high- and low-risk groups in the TCGA cohort was examined to investigate their functions in glioma. Further functional analysis was performed by GSEA using the HALLMARK (**Figure 8A**), KEGG (**Figure 8B**), and Reactome (**Figure 8C**) databases. Examination of the top 30 pathways showed that the TP53 signaling pathway was enriched in all three databases. Many of the enriched signaling pathways in the three databases were closely related to the cell cycle and DNA damage repair. Other enriched pathways were associated with tumor progression, including the IL6/JAK/STAT3, KRAS, and PI3K/AKT/mTOR signaling pathways, which corresponded to the higher mutation rate of EGFR and PTEN in high-risk patients mentioned above.

### Identification of an RTK Pathway Inhibitor With Significant Inhibitory Activity in High-Risk Patients

**Figure 9:**
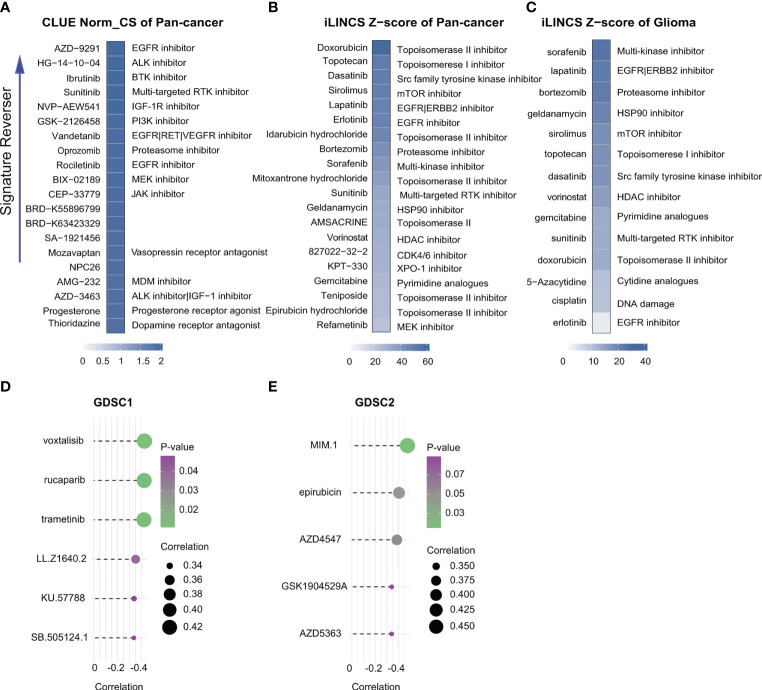
Drug connectivity analysis using alteration-specific transcriptional (CLUE and iLINCS). Pan-cancer in CLUE **(A)**, pan-cancer in iLINCS **(B)**, and glioma in iLINCS **(C)** identifying 20 compounds that enhance or reverse the signature (highlighted with documented mechanisms). Compounds showing negative correlations with AUC values and FRG scores for glioma cells were identified from GDSC1 and GDSC2. **(D, E)**.

Correlation analysis identified six GDSC1-derived compounds, namely, voxtalisib, rucaparib, trametinib, LL.Z1640.2, KU.57788, and SB.505124.1, and five GDSC2-derived compounds, namely, MIM.1, epirubicin, AZD4547, GSK1904529A, and AZD5363 (**Figures 9 D, E**). Although these candidate compounds showed high drug sensitivities in the high-risk group, this evidence is insufficient for supporting the conclusion that they are effective for treating gliomas. Therefore, iLINCs and CLUE were used to determine which compounds could counteract cancer- or glioma-specific gene expression (i.e., gene expression was increased in tumor tissues but decreased in response to treatment with the compound). We submitted 150 genes with the highest and lowest expression, respectively, to CLUE and 150 genes with the lowest expression to iLINCs. The CLUE results (cMAP scores) were subsequently normalized. The top 20 compounds with the potential to reverse the action of FRGs in high-risk patients are listed in **Figures 9A–C**. Of these, only epirubicin was found to match the GDSC2 results directly, with the other compounds showing similar matches. Thus, we concluded that patients with FRG scores may benefit more from an RTK pathway inhibitor, either by direct inhibition of the RTK or indirect inhibition through targeting downstream effectors such as the PI3K or MAPK pathways.

## Discussion

Ferroptosis is a recently described iron-dependent form of PCD, differing from other PCD types such as apoptosis and necroptosis (37). The ferrDB database contains information on all genes linked to ferroptosis and provides the latest resources for research (21). Five ferroptosis-related gene signatures are currently recognized as related to survival in glioma. Gene signatures have been constructed from the investigation of 60-80 candidate genes (9, 38, 39) identified by previous publications. When Zhou and Sun downloaded 173 and 303 FRGs, respectively, from the ferrDB database (40, 41), the overall number of candidate genes was relatively small. The numbers and functional classification of FRGs in the ferrDB are constantly updated as new information becomes available, and new analyses of ferroptosis signatures for glioma have become more necessary. In this study, we applied the latest data including 388 driver, suppressor, and marker genes from the ferrDB database (**Figure 2A**). At the same time, before the conventional screening of genes using univariate and multivariate Cox regression analysis, we collected data on CRISPR-screened glioma-associated genes from the DepMap portal (**Figure 2A**) and investigated their intersection with the FRGs. This led to the identification of 10 cancer-essential FRGs (ISCU, NFS1, MTOR, EIF2S1, HSPA5, AURKA, RPL8, LONP1, RRM2, and CHMP6) which were closely related to ferroptosis and essential for glioma cell proliferation and survival. The use of the CRISPR screening technique explored potential cancer-essential FRGs, which may shed light on the discovery of targetable FRGs in other tumors or diseases for research.

After multivariate and LASSO analyses, seven cancer-essential FRGs (ISCU, NFS1, MTOR, EIF2S1, HSPA5, AURKA, and RPL8) were used to construct the risk model (**Figures 2B–D**). This model was shown to have stable prognostic prediction capability in both the training and validation cohorts (**Figures 3A–D**) and the subgroups based on clinical features in the validation cohort (**Figure 5**). Further analysis showed that the risk score independently predicted survival in glioma and was effective for predicting both OS and progression-free survival (**Figures 7C–D**). These findings strongly implied that these seven cancer-essential FRGs play important roles in many aspects of glioma development and progression. We further examined the classifications of the risk model genes, finding that three (ISCU, NFS1, and HSPA5) were suppressors of ferroptosis, two (EIF2S1 and AURKA) were marker genes, RPL8 was classified as a driver gene, and MTOR was classified as both a driver and a suppressor gene. Thus, most of the risk model genes were ferroptosis suppressors. On the other hand, all the risk model genes had been identified by CRISPR screening as glioma-associated in glioma cell lines and thus would be involved in the promotion of proliferation (22). In terms of single gene functions, ISCU encodes part of the iron-sulfur (Fe-S) cluster scaffold that synthesizes Fe-S clusters in the mitochondria (42). It has been found that ISCU is targeted by miR-210-3p to block the growth and migration of glioma cells *in vitro* (43). NFS1 encodes several proteins involved in supplying S derived from cysteine to Fe-S clusters and inhibits cysteine transport, triggering ferroptosis and reducing tumor cell growth (44). HSPA5 is involved in protein folding in the endoplasmic reticulum (45), assists in fungal infection of epithelial cells (46), and has been implicated in both proliferation and apoptosis (47). Its expression is promoted by iron in the nasal epithelium (48–50) and is used as a target for >treating chemo-resistant cancers (51). mTOR plays a major role in various metabolic processes, mediating the actions of a variety of hormones, growth factors, and other signaling molecules (52–54). The risk model gene AURKA is involved in cell cycle modulation (55, 56), specifically during mitosis (57), and also regulates phosphorylation of p53/TP53, affecting cancer development (58). In terms of somatic mutation, we found that the frequency of the IDH1 mutation was greater in the low-risk group, in contrast to the higher mutation rates seen in both PTEN and EGFR in the high-risk group (**Figures 6A, B**), suggesting that the mutation frequencies in those genes may have different effects on ferroptosis. Previous studies have shown that IDH-mut glioma patients had a better prognosis than those with wild-type IDH, across all WHO grades (11, 40), that EGFR amplification was correlated with poor prognosis (59), and that PTEN mutations could promote the infiltration of immunosuppressive tumor-associated macrophages (TAMs) (60). This suggests that FRGs represent good indicators of malignancy-associated glioma characteristics. The analysis of immune cell infiltration showed higher scores for memory B cells, CD8^+^ T cells, CtD4^+^ naïve and memory T cells, Tregs, neutrophils, and M2 macrophages in the high-risk group. These results indicated that the patients in the two risk groups have completely different immune microenvironments. Studies have shown that CD4+ T cells, B cells, CD8+ T cells, neutrophils, and M2 macrophages play important roles in the occurrence and malignant progression of glioma (61–63) and that Tregs are closely associated with significant immune suppression in the tumor (64), thus supporting the results of our study. The expression of immune checkpoints was significantly increased in the high-risk group except for VTCN1, indicating a close relationship with glioma immune escape. Tumor immunotherapy is currently an attractive treatment and has achieved good results in other cancers, such as lung cancer. Unfortunately, these have yielded little success clinically, and all clinical trials of immunotherapy have failed. The establishment of the current model may be useful for screening patients for glioma immunotherapy. The enrichment analysis also showed that the model genes were associated with DNA replication and repair and the cell cycle, as well as several signaling pathways closely related to tumor progression (**Figures 8A–C**). Indeed, the high degree of DNA damage and poor repair capacity seen in glioma cells is a trademark feature of cancer and is a determining factor in the growth of tumor cell populations. The gene signature was found to be highly enriched in the cell cycle and DNA damage repair functions; thus, we speculate that these genes play significant parts in cell cycle regulation and tumor development and progression. This suggests an explanation for the influence of FRGs on patient responses, although there may be other reasons also. These genes are thus relevant for the diagnosis and treatment of cancer.

The treatment of malignant brain cancers is challenging. This is especially true of late-stage glioma which is resistant to most traditional types of therapy (65). Recently, an association between ferroptosis-related signatures and drug screening was found in liver cancer (66) and the potential of enhancing treatment efficacy by inducing ferroptosis has attracted attention. Although studies on glioma have focused on the use of ferroptosis to predict prognosis, none have investigated potential drugs (9, 36, 37). Here, we used GDSC1, GDSC2, iLINCS, and CLUE to identify molecules that might reverse the tumor signature, observing that patients with high FRG scores may benefit more from treatment with an RTK pathway inhibitor, either by direct inhibition of the RTK or indirect inhibition by targeting downstream effectors such as the PI3K or MAPK pathways (**Figure 9**). For primary glioblastoma, bevacizumab (a humanized monoclonal antibody against VEGF-A to block angiogenesis) combined with Stupp is a currently widely used strategy which, although it prolongs the median progression-free survival, does not appear to influence the OS. Using the gene signature for screening patients before deciding on the treatment regimen may improve the efficacy of personalized treatment of glioma.

The study has several limitations. Firstly, ferroptosis-associated pathways are not well understood, and it is possible that the signature genes may function in other pathways as well, for example, in autophagic or immune pathways. Secondly, a major limitation is that we used a public database rather than our own samples; we intend to collect our own glioma samples to further verify the reliability of the research results. Finally, further basic and clinical research is required for verification of the signature application, which will be an important direction of our future research.

To summarize, we identified a seven-gene cancer-essential FRG signature for the prediction of glioma patient prognosis. The signature was found to have an excellent prognostic capability and was utilized for calculating risk scores and drug identification. The model thus has potential in both the diagnosis and management of glioma.

## Data Availability Statement

The original contributions presented in the study are included in the article/**Supplementary Material**. Further inquiries can be directed to the corresponding author.

## Author Contributions

DY and XYW designed the study and analyzed the data. WW, XR, XSW, and JSL collected the data. DY, XR, JF, and JBL drafted and revised the manuscript. XDR, HZ, LC, TL, CZ, and SY revised the images. DY, XYW, and XY revised the manuscript. All authors contributed to the article and approved the submitted version.

## Funding

This work was supported by the grants from the National Natural Science Foundation of China (No.81872063), Beijing-Tianjin-Hebei Basic Research Cooperation Project (No. 19JCZDJC64200) and Guangxi Natural Science Foundation of China (2018JJA140739).

## Conflict of Interest

The authors declare that the research was conducted in the absence of any commercial or financial relationships that could be construed as a potential conflict of interest.

## Publisher’s Note

All claims expressed in this article are solely those of the authors and do not necessarily represent those of their affiliated organizations, or those of the publisher, the editors and the reviewers. Any product that may be evaluated in this article, or claim that may be made by its manufacturer, is not guaranteed or endorsed by the publisher.
